# Do MAFLD Patients with Harmful Alcohol Consumption Have a Different Dietary Intake?

**DOI:** 10.3390/nu14071335

**Published:** 2022-03-23

**Authors:** Sara Policarpo, Sofia Carvalhana, Ana Craciun, Ricardo Rios Crespo, Helena Cortez-Pinto

**Affiliations:** 1 Laboratório de Nutrição, Faculdade de Medicina, Universidade de Lisboa, 1649-028 Lisboa, Portugal; sofiacarvalhana@msn.com (S.C.); hlcortezpinto@gmail.com (H.C.-P.); 2 Serviço de Dietética e Nutrição, Centro Hospitalar Universitário Lisboa Norte, E.P.E., 1649-035 Lisboa, Portugal; 3 Departamento de Gastrenterologia, Centro Hospitalar Universitário Lisboa Norte, E.P.E., 1649-035 Lisboa, Portugal; aanacraciun@gmail.com (A.C.); rr.crespo91@gmail.com (R.R.C.); 4 Clínica Universitária de Gastrenterologia, Faculdade de Medicina, Universidade de Lisboa, 1649-028 Lisboa, Portugal

**Keywords:** dietary inflammatory index, sugar, fat, steatosis, advanced fibrosis

## Abstract

The term metabolic-associated fatty liver disease (MAFLD) has been proposed to define positively fatty liver disease in the form associated with metabolic risk factors. The aim of this study was to assess the dietary intake of MAFLD and explore a possible relationship between its inflammatory characteristics (assessed by Dietary Inflammatory Index—DII^®^), the degree of liver fibrosis (assessed by transient elastography), and the amount of alcohol intake. MAFLD patients were included (*n* = 161) and were classified, according to the amount of alcoholic intake, as MAFLD without alcohol intake (*n* = 77) and MAFLD with alcohol intake (*n* = 84), with 19 presenting harmful alcoholic consumption. Dietary intake was 1868 ± 415 kcal/day and did not present differences in energy or nutrient intake based on the presence of metabolic comorbidities. Patients with MAFLD and alcohol intake consumed significantly more energy and presented a tendency for higher intake of carbohydrates and sugar. Patients with harmful alcohol intake presented a higher intake of total fat and cholesterol compared with moderate alcohol intake. There were no differences in DII^®^ based on fibrosis severity or the amount of alcohol consumption. This work contributes to the characterization of baseline dietary intake in MAFLD patients, paving the way to design more suited dietary interventional trials.

## 1. Introduction

Non-alcoholic fatty liver disease (NAFLD) is a multisystemic disease that occurs in association with the metabolic syndrome and in the absence of harmful alcohol intake. The cornerstone of NAFLD treatment is lifestyle changes, since there is no pharmacological treatment available [1,2,3].

Recently, an international panel of experts proposed a change in nomenclature to metabolic dysfunction-associated fatty liver disease (MAFLD) [4]. This new designation emphasizes the heterogeneity in the underlying causes and manifestations, clinical course and outcome, also avoiding a definition based on a negative. According to the authors, it is more suitable to label the group of liver diseases associated with metabolic dysfunction [4,5]. In addition, regarding nutritional status, the positive criteria for MAFLD includes lean patients, who often have body composition alterations that can affect the progression to a more severe form of the disease [6,7]. Still, the proposed definition remains controversial [8].

Diet influences liver fat content [9], and although numerous nutritional approaches have proven effective in promoting weight loss in NAFLD, the Mediterranean diet has been more robustly associated with better metabolic outcomes, improvement in insulin resistance, and better lipid profile, independently of weight loss [3,10,11,12]—possibly because of the diet’s anti-inflammatory properties, which can contribute to mitigate hepatic stress [12,13,14,15].

Since the human diet consists of foods and nutrients that can be predominantly pro-inflammatory or anti-inflammatory, resulting in different levels of inflammatory biomarkers [16], a dietary pattern approach acknowledges the complex interactions between dietary components, and it has advantages over individual foods or nutrients when studying associations with disease [17].

The Dietary Inflammatory Index (DII^®^) is a literature-derived, population-based index that was developed to predict the inflammatory potential of diet [18]. The DII^®^ has been validated with various inflammatory markers. A more pro-inflammatory dietary pattern has been associated with higher mortality risk [19,20,21].

A less pro-inflammatory diet has been demonstrated to be associated with less liver fat [22,23,24]. However, studies were conducted mostly with non-invasive scores of liver fat, and there are no data, to our knowledge, regarding DII^®^ and stages of liver fibrosis, the latter strongly implicated in the outcome of fatty liver diseases. In addition, different degrees of alcohol intake can, possibly, have different inflammatory properties.

Since MAFLD has only recently been proposed, with scarce information regarding the impact of dietary pattern in the development and progression of fatty liver disease [25], the aim of the present study was to characterize the dietary intake in MAFLD patients, focusing on the possible relationship between dietary pattern—assessed by DII^®^, and degree of liver fibrosis, as well as on the effect of harmful alcohol consumption in this equation.

## 2. Materials and Methods

This is a cross-sectional study, conducted at the outpatient Hepatology Clinic of a tertiary university hospital, where patients are referred due to suspected fatty liver disease by general practitioners or by other medical specialists within the hospital.

### 2.1. Participants

During a six-month period, patients with a first appointment for suspected fatty liver disease were included in the analysis. Nutritional assessment, conducted by a single dietitian, included anthropometric and dietary data. Clinical data were recorded by a group of trained hepatologists, who used a standardized protocol to minimize bias on data collection. All data were collected in the first appointment. Patients who were unable to complete full nutritional assessment (detailed in following sections) or who did not present liver steatosis (detailed in Section 2.4) were excluded.

On the fasting state (12 h fasting), blood samples were collected in an accredited laboratory using standard methodology for the determination of fasting glucose (Hexokinase UV test, Cobas 8000 c702 (Roche Diagnostics, Mannheim, Germany)), insulin (ECLIA sandwich assay, Cobas 8000 e801 (Roche Diagnostics, Mannheim, Germany)), HbA1c (Boronate affinity HPLC, Premier Hb9210 (A. Menarini Diagnostics, Florence, Italy)), total cholesterol (Enzymatic colorimetric method, Cobas 8000 c702 (Roche Diagnostics, Mannheim, Germany)), triglycerides (TG) (Enzymatic colorimetric method, Cobas 8000 c702 (Roche Diagnostics, Mannheim, Germany)), HDL-cholesterol (HDL-c) (Homogeneous enzymatic colorimetric assay, Cobas 8000 c702 (Roche Diagnostics, Mannheim, Germany)), and liver enzymes (AST—aspartate aminotransferase and ALT—alanine aminotransferase; Enzymatic IFCC modified—without pyridoxal phosphate activation, Cobas 8000 c702 (Roche Diagnostics, Mannheim, Germany) and GGT—gamma glutamyl transferase; Enzymatic-Szasz, Cobas 8000 c702 (Roche Diagnostics, Mannheim, Germany)). LDL-cholesterol (LDL-c) was calculated using the Friedewald formula in mg/dL (c-LDL = CT − (c-HDL + TG/5)).

### 2.2. Anthropometric Parameters

Anthropometric data were collected with participants wearing light clothes and bare feet. Weight was measured using a calibrated scale and height was assessed using a stadiometer. Waist circumference (WC) was measured halfway between the inferior rib and the iliac crest. Body mass index (BMI) was defined as an individual’s weight in kilograms divided by the square of height in meters (kg/m^2^). Body fat mass was determined using bioelectrical impedance analysis (BIA—Biodynamics BIA 450 Bioimpedance Analyzer).

### 2.3. Dietary Intake and DII^®^

A validated food frequency questionnaire developed for the national population (FFQ) was applied by a trained investigator on the first appointment. The FFQ is a semi-quantitative questionnaire comprising 89 food items, distributed by 8 categories (dairy; eggs, meat and fish; fat; cereals; sweat and pastries; vegetables and legumes; fruits and drinks), ranging from daily to monthly frequency and allows the quantification of energy and 44 nutrients, reflecting the dietary intake of the previous year [26].

Shivappa et al. [16] documented that a total of 45 specific foods and nutrients were associated with one or more inflammatory markers: IL-1b, IL-6, TNF-α, or CRP. They scored the inflammatory potential for each food parameter according to its relation to inflammatory markers: increased, decreased or no effect. Then, based on 11 data sets from 11 countries worldwide, they calculated the mean and standard deviation for each of the 45 food parameters. Subsequent studies acknowledged that it is possible to assess DII^®^ with less food items [18].

Dietary data derived from the FFQ were used to calculate DII^®^ scores for all participants. As documented previously [18], we calculated DII^®^ score based on 28 food parameters from the FFQ: energy, protein, carbohydrates, fiber, total fat, saturated fat, mono-unsaturated fat, polyunsaturated fat, omega-3, omega-6, trans fat, thiamine, folic acid, niacin, riboflavin, vitamin B12, vitamin B6, vitamin C, vitamin A, vitamin D, vitamin E, iron, selenium, magnesium, zinc, alcohol, and caffeine. To calculate the DII^®^ score for each participant, a z score for a given food parameter was calculated by subtracting the “standard global mean” from the amount consumed by each participant and dividing this value by the “global standard deviation”. Then, this value was converted to a centered percentile score. For each participant, this score was then multiplied by the respective food parameter effect score [25]. The overall DII^®^ score was calculated for each participant by summing up all DII^®^ scores from each calculated FFQ item. A higher and positive DII^®^ score indicates a more inflammatory diet, and a lower and negative DII^®^ score indicates a less inflammatory diet. The DII^®^ score was categorized into quartiles and coded as more anti-inflammatory or pro-inflammatory based on the patients’ distribution.

### 2.4. Assessment of MAFLD

On the initial evaluation of patients with suspected MAFLD, the presence of liver steatosis on ultrasound, which uses sound waves to evaluate the size and shape of the liver, as well as blood flow through the liver, was assessed, and a transient hepatic elastography (Fibroscan^®^) evaluation, which measures, in the middle of the liver, the movement of the liver caused by ultrasound wave and allows the determination of its stiffness (or elasticity), was conducted. Fibroscan^®^ allows the determination of Controlled Attenuation Parameter (CAP) as an indicator of the degree of steatosis, and it measures liver stiffness measurement (LSM) as an indicator of liver fibrosis [27,28,29]. The values chosen to indicate steatosis as absent (S0), mild (S1), moderate (S2), and severe (S3) were: S0 < 236 dB/m, S1 ≥ 236 dB/m, S2 ≥ 270 dB/m, and S3 ≥ 302 dB/m [30]. Fibrosis was defined as absent (F0), mild (F1 ≥ 5.5 kPa), moderate (F2–F3 ≥ 7 kPa), and advanced (F4 ≥ 10.5 kPa) [29].

The patient was considered as having MAFLD if liver steatosis was present on ultrasound, plus the presence of one of three factors: overweight (BMI ≥ 25 kg/m^2^) or type 2 diabetes (T2D if fasting glucose levels, HbA1c ≥ 6.5% or) or lean BMI (BMI < 25 kg/m^2^) plus the presence of two metabolic risk abnormalities (waist circumference ≥ 102/88 cm in Caucasian men and women, blood pressure ≥ 130/85 mmHg or specific drug treatment, plasma triglycerides ≥ 150 mg/dL (≥1.70 mmol/L) or specific drug treatment, plasma HDL-cholesterol < 40 mg/dL (<1.0 mmol/L) for men and <50 mg/dL (<1.3 mmol/L) for women or specific drug treatment, prediabetes (i.e., fasting glucose levels 100 to 125 mg/dL (5.6 to 6.9 mmol/L), or 2 h post-load glucose levels 140 to 199 mg/dL (7.8 to 11.0 mmol), or HbA1c 5.7% to 6.4% (39 to 47 mmol/mol)), homeostasis model assessment of insulin resistance score ≥ 2.5 or plasma high-sensitivity C-reactive protein level > 2 mg/L) [4]. MAFLD patients were further classified as MAFLD with alcohol intake and MAFLD without alcohol intake, according to alcoholic beverage consumption, as previously described [5].

Metabolic syndrome was considered when 3 of 5 criteria were present [31]: elevated waist circumference ≥ 94/88 cm in Caucasian men and women, elevated blood pressure ≥ 130/85 mmHg or specific drug treatment, elevated plasma triglycerides ≥ 150 mg/dL or specific drug treatment, reduced plasma HDL-cholesterol < 40 mg/dL for men and <50 mg/dL for women or specific drug treatment, fasting glucose ≥ 100 mg/dL or specific drug treatment.

Patients who presented positivity to hepatitis B virus surface antigen; positivity to anti-hepatitis C virus; other type of liver diseases (i.e., primary biliary cholangitis, autoimmune hepatitis, primary sclerosing cholangitis, Wilson’s disease, hemochromatosis, or a1-antitripsin deficiency); and treatment with potentially steatogenic drugs such as steroids, high-dose estrogen, tamoxifen, methotrexate, or amiodarone were classified as having dual etiology [4].

Patients were further classified, according to the amount of alcoholic consumption, as MAFLD without alcohol intake and MAFLD with alcohol intake. Those who presented alcohol intake higher than 20 g/day for women and 30 g/day for men were considered further as MAFLD with harmful alcohol intake and compared with those with moderate intake (alcohol intake below 20 g/day for women and 30 g/day).

### 2.5. Statistical Analysis

Continuous variables were summarized using mean and standard deviation (SD). Categorical data are represented using frequency tables. Unpaired *t*-tests (Student’s *t* test or one-way ANOVA) were used to compare distribution across continuous variables. When variables did not follow a normal distribution, as assessed by Shapiro–Wilk test and inspection of distribution graphs, the non-parametric test alternative was used (Mann–Whitney or Kruskal–Wallis) Chi-Squared test was used for categorical variables to test differences between different groups. To verify the association between continuous variables, Pearson’s correlation coefficient was used. The odds ratio (OR) and 95% CI summarizing the association between DII^®^ and severe steatosis and advanced liver fibrosis were calculated. The following variables were adjusted in the logistic regression model: age, BMI, metabolic syndrome, dyslipidemia, HBP, diabetes, and sex. A *p*-value < 0.05 was used as the cut-off for statistical significance. All statistical analysis was conducted using SPSS^®^ (IBM SPSS Statistics 26).

## 3. Results

**Figure 1:**
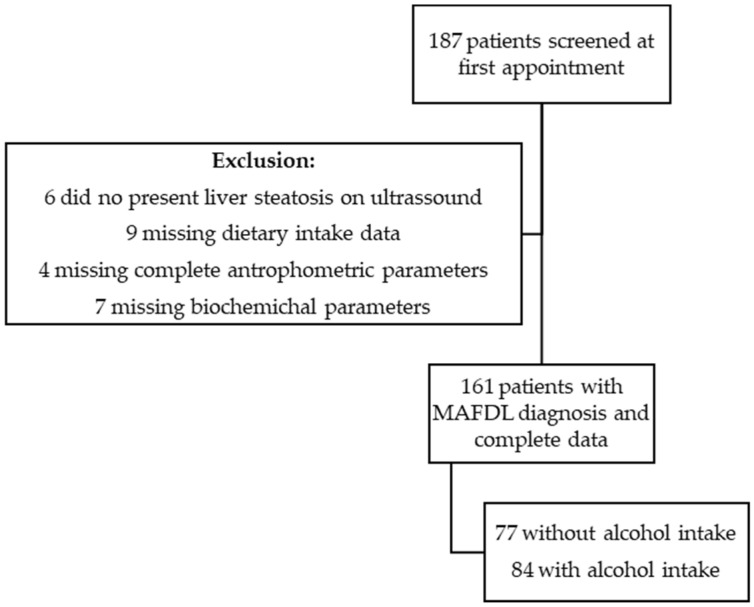
Flowchart of patient’s inclusion.

From the 187 patients screened, 161 had complete nutritional and clinical assessment, and MAFLD diagnosis was established. None of the patients presented dual etiology. From the 161 patients, 47.8% (*n* = 77) presented MAFLD without alcohol intake and 52.2% (*n* = 84) presented MAFLD with alcohol intake; from these, 22.6% (*n* = 19) presented harmful alcoholic consumption (Figure 1).

Patients were mostly males (59.6%; *n* = 96); 91.9% (*n* = 148) presented overweight (BMI ≥ 25 kg/m^2^). When comparing MAFLD patients with or without alcohol intake, there was a trend for patients without alcohol intake to present a higher prevalence of high blood pressure and metabolic syndrome (Table 1).

Male patients were significantly associated with the presence of MAFLD with alcohol intake (OR: 2.286 (IC95%: 1.505–3.471); *p* < 0.001). No association between BMI category (BMI < 25 kg/m^2^ vs. BMI ≥ 25 kg/m^2^) and alcohol intake was found.

MAFLD patients with metabolic syndrome were older (61.0 ± 9.7 vs. 50.7 ± 13.4 years; *p* < 0.001) and presented higher mean levels of BMI (32.5 ± 5.4 vs. 29.3 ± 4.7 kg/m^2^; *p* < 0.001) and waist circumference (110 ± 12.1 vs. 103.9 ± 9.8 cm; *p* = 0.011).

Patients with moderate alcohol intake did not present significant differences in mean age, BMI, or metabolic comorbidities prevalence, when compared with patients with harmful alcohol intake.

### 3.1. Dietary Intake and DII^®^

Patients reported a mean dietary caloric intake of 1868 ± 415 kcal/day. Patients with any alcohol intake presented higher intake of calories, higher median global daily intake of red meat (35.7 vs. 46 g/day; *p* = 0.024), pastries (13 vs. 15 g/day; *p* = 0.038), added sugar (5.8 vs. 7.6 g/day; *p* = 0.019), and sugar-sweetened beverages (26.5 vs. 48.2 mL/day; *p* = 0.045). This group with any alcohol intake also presented a tendency for higher intake of carbohydrates and sugar (Table 2).

Comparing further only the patients with alcohol intake, those with moderated alcohol intake when compared with harmful alcohol intake presented higher median global daily intake of total fat (76.8 vs. 63.1 g/day; *p* = 0.008), saturated fat (22.2 vs. 18.7 g/day; *p* = 0.032), MUFA (35.3 vs. 28.7 g/day; *p* = 0.09), PUFA (13.1 vs. 10.4 g/day; *p* = 0.007), and cholesterol (295.7 vs. 247.7 mg/day; *p* = 0.027), even though there was no significant difference in mean caloric, protein, or carbohydrates intake between groups. In addition, there was a tendency for patients with moderated alcohol intake to present higher median intakes of iron (14.5 vs. 12.2 mg/day; *p* = 0.068), magnesium (337 vs. 289 mg/day; *p* = 0.077), and omega-6 fatty acids (9.4 vs. 8.0 g/day; *p* = 0.074) compared with those with harmful alcohol intake. Patients with harmful alcohol intake presented lower median daily intake of dairy (62.0 vs. 131.3 mL/day; *p* = 0025) and chicken (27.4 vs. 40.4 g/day; *p* = 0.030) when compared with the group with moderated alcohol intake.

Patients with harmful alcohol intake presented a higher mean energy intake compared with non-drinkers (2256 ± 929 kcal; *p* = 0.004) with no other nutritional difference.

Based on BMI, there were no differences in dietary intake in the group of patients with no alcohol intake and moderated alcohol intake. In patients with harmful alcohol intake, there was a significant difference in sugar intake from pre-obese patients and obese patients (122.8 g/day vs. 70.7 g/day; *p* = 0.028) but not between lean patients and the remaining.

MAFLD patients with metabolic syndrome did not present any significant differences in dietary intake compared with the remaining group.

Mean DII^®^ was 0.0042 (−3.92; 4.58); 50.3% (*n* = 81) presented a predominantly anti-inflammatory DII^®^ and 49.7% (*n* = 80) presented a more pro-inflammatory DII^®^. There were no differences in BMI category, or the presence of metabolic syndrome based on DII^®^ quartiles.

### 3.2. Liver Disease

Patients presented a mean CAP of 298.9 ± 62.9 dB/m, with 56.1% (*n* = 88) presenting severe steatosis, 48.7% (*n* = 78) presenting moderate fibrosis, and 21.3% (*n* = 34) presenting advanced fibrosis.

Based on BMI category, there were no significant differences in mean CAP, but mean LSM differed significantly between BMI categories, with obese patients presenting higher mean LSM (10.0 ± 6.8 kPa) compared with lean patients (5.9 ± 1.9 kPa; *p* = 0.038) and pre-obese patients (7.0 ± 4.1 kPa; *p* = 0.004).

MAFLD patients with metabolic syndrome presented higher mean levels of CAP (316.3 vs. 282.3 dB/m; *p* = 0.001) and fibrosis (10.1 vs. 6.6 kPa; *p* < 0.001) compared with the remaining.

Some metabolic factors were associated with a higher risk of presenting advanced fibrosis: namely, the presence of diabetes (OR: 7.250 (IC: 3.014–17.441); *p* < 0.001), high blood pressure (OR: 2.369 (IC: 1.024–5.480); *p* = 0.040), metabolic syndrome (OR: 4.333 (IC: 1.820–10.317); *p* = 0.001), and BMI ≥ 30 kg/m^2^ (OR: 2.531 (IC: 1.109–5.574); *p* = 0.025).

Mean CAP and LSM did not present differences from MAFLD patients with alcohol intake, regardless of the amount consumed, compared with MAFLD patients without alcohol intake. The prevalence of severe steatosis or advanced fibrosis between groups also did not present significant differences. When adjusting for baseline BMI, in the group of patients with moderate alcohol intake, there was a significant difference in mean LSM, with obese patients presenting higher mean LSM (9.6 ± 6.1 kPa) compared with lean patients (5.4 ± 1.3 kPa; *p* = 0.032) and pre-obese patients (6.4 ± 2.9 kPa; *p* = 0.007). There was no significant difference in mean CAP or LSM, based on BMI in the group of patients with no alcohol intake and with harmful alcohol intake. The prevalence of severe steatosis or advanced fibrosis between groups of alcohol intake based on BMI category also did not present significant differences.

### 3.3. Dietary Intake, DII^®^, and Liver Disease

Patients did not present significant differences in caloric or nutrient intake (protein, CHO, total fat and fractions, fiber, sugar, and caffeine) based on the presence of metabolic comorbidities or metabolic syndrome.

Patients with severe steatosis presented higher median intakes of simple carbohydrates (109.8 vs. 85.0 g/day; *p* = 0.046) and a tendency to lesser folic acid intake (compared with the remaining). When compared with patients with mild steatosis, patients with severe steatosis presented higher median intake of added sugar (8.0 vs. 1.0 g/day; *p* = 0.001) and a tendency for a higher median intake of pastries (9.4 vs. 5.8 g/day; *p* = 0.083) and sugar sweetened beverages (20.9 vs. 10.0 mL/day; *p* = 0.081).

Patients with advanced fibrosis presented lower median intake of total fat (65.4 vs. 77.3 g/day; *p* = 0.024), saturated fat (18.2 vs. 22.5 g/day; *p* = 0.014), and MUFA (30.4 vs. 35.6 g/day; *p* = 0.025) and a tendency for lower caloric intake (1687 vs. 1927 kcal/day; *p* = 0.014) and PUFA intake (11.4 vs. 13.1 g/day; *p* = 0.014) compared with the remaining. In addition, they presented lower median intakes of riboflavin (1.4 vs. 1.7 mcg/day; *p* = 0.022), calcium (575 vs. 778 mg/day; *p* = 0.009), and zinc (8.9 vs. 10.6 mg/day; *p* = 0.017).

**Figure 2:**
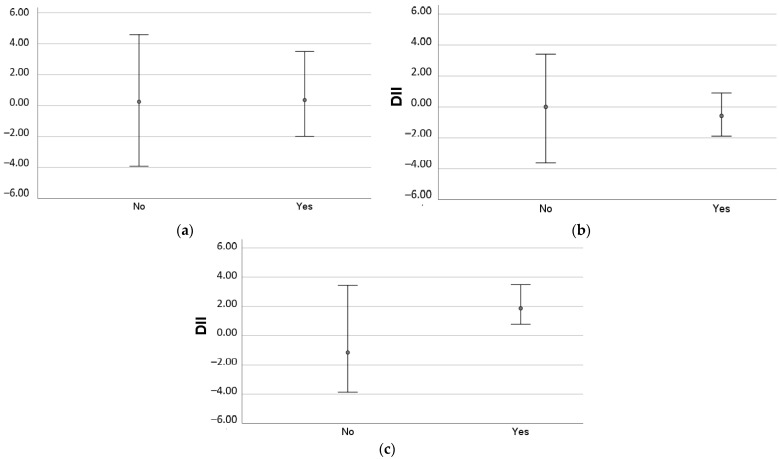
Mean DII based on the presence of advanced fibrosis in MAFLD patients (**a**) without alcohol intake; (**b**) with moderate alcohol intake; (**c**) with harmful alcohol intake.

No correlation was found between raw and quartiles of DII^®^ and liver enzymes, liver steatosis (measured by CAP), or LSM.However, in the group of patients with no alcohol intake, those who presented a more pro-inflammatory DII^®^ more frequently had severe steatosis (87.5% vs. 12.5%), with a predominantly pro-inflammatory diet associated with a four-fold increase in the odds of having severe steatosis (OR: 4.000 (95%CI 1.129–14.175); *p* = 0.026). In patients with harmful alcohol intake, a more pro-inflammatory DII^®^ was associated with a higher degree of fibrosis, with all patients with advanced fibrosis presenting a pro-inflammatory DII^®^ (*p* = 0.027) (Figure 2a–c)).

In a multinomial logistic regression model, in patients that did not present alcohol intake, after adjustment, a more pro-inflammatory diet maintained its association with a higher probability of presenting severe steatosis (OR: 4.789 [95%CI 1.102–20.807]; *p* = 0.037).

## 4. Discussion

The aim of this study was to examine the dietary intake of MAFLD patients and explore possible links with DII^®^ and the degree of liver fibrosis. To the best of our knowledge, this is the first study conducted assessing DII^®^ in MAFLD using transient hepatic elastography.

Although in prospective studies of NAFLD patients, a higher DII^®^ has been associated with higher probability of steatosis, based on non-invasive scores, such as the fatty liver index [22,24], as well as with higher risk of metabolic syndrome [32], in this cross-sectional study, with MAFLD patients, we did not find such correlation nor a significant correlation with BMI. A recent study comparing 968 MAFLD patients with 964 controls found that a more pro-inflammatory dietary score was associated with a higher risk of MAFLD [33], although no investigation was conducted on the severity of the disease. In our study, we observed that MAFLD patients with no alcohol intake had a more pro-inflammatory diet, and this was associated with a higher likelihood of having severe steatosis. This association prevailed after adjustment for the major metabolic comorbidities. In addition, all patients with harmful alcohol intake and advanced fibrosis presented a pro-inflammatory DII^®^.

In this study, the inflammatory component of the diet appears to be not so relevant in patients with alcohol intake, independently of the amount consumed. In patients with NAFLD, a moderate alcohol consumption is allowed, and with MAFLD, there is currently no defined threshold for alcohol intake [4]. Although the impact of chronic alcohol consumption on the liver is very well demonstrated [34], the health benefits of moderate alcohol consumption, compared with abstainers and heavy drinkers, is an ongoing debate, with findings being inconsistent and varying depending on the studied outcome [35]. In this study, the inflammatory degree of the diet was of most relevance in non-drinkers. It is possible that in the absence of alcohol consumption, diet becomes more relevant to the risk of developing fatty liver disease. However, all patients with harmful alcohol intake and advanced fibrosis presented a more pro-inflammatory DII^®^, suggesting that in this circumstance, alcohol and a pro-inflammatory diet may have a synergistic effect in the progression of liver disease.

We also found that globally, patients with MAFLD present an unbalanced dietary intake, with higher intakes of added sugar, sugar-sweetened beverages, and pastries. In our study, similar to the results of a recent meta-analysis [36] that included 60 eligible studies with 100,621 patients, we did not find any correlation between nutrient intake and the degree of steatosis and fibrosis.

This study has some limitations. The reduced sample size of patients with harmful alcohol intake does not allow us to extrapolate the data and can possibly limit the amount of significance achieved. In addition, the use of dietary scores, such as the DII^®^, although useful to compare different populations, is based on a selection of specific aspects of the diet and cannot describe with complete accuracy the overall dietary pattern [37]. Since MAFLD is a new concept, harmful alcohol intake could also be incorporated in dual etiology [8]. However, in the present study, the contribution of this aspect was within the aim of the study, and since the dietary intake of these patients can have some differences from the NAFLD patients, it may be easier to evaluate what may represent the additive effect of harmful alcohol intake.

In the patient-centered, multidisciplinary management, recommended for these patients [38], an accurate knowledge of the dietary intake and is essential to tailor a successful lifestyle intervention. The findings of this study contribute to the characterization of baseline dietary intake and inflammatory degree of the diet in MAFLD patients, paving the way to design more suited dietary interventional trials.

## Figures and Tables

**Table 1:** Baseline patient characteristics.

			MAFLD Patients (*n* = 161)	MAFLD without Alcohol (*n* = 77)	MAFLD with Alcohol (*n* = 84)	*p*-Value *
Sex (%)		Male	59.6 (96)	42.9 (33)	75.0 (63)	<0.001
		Female	40.4 (65)	57.1 (44)	25.0 (21)	
Age (years)			55.9 ± 12.7	55.2 ± 13.3	56.5 ± 12.2	0.534
Weight (kg)			86.3 ± 16.5	83.5 ± 16.9	89.0 ± 15.7	0.035
BMI (kg/m^2^)			30.9 ± 5.3	30.9 ± 5.3	30.8 ± 5.3	0.909
BMI	Normal (<25 kg/m^2^)		8.1 (13)	6.5 (5)	9.5 (8)	0.732
	Pre-obese (≥25–29.9 kg/m^2^)		42.2 (68)	41.6 (32)	42.9 (36)	
	Obese (≥30 kg/m^2^)		49.7 (80)	51.9 (40)	47.6 (40)	
Waist circumference (cm)			102.9 ± 10.8	100.9 ± 9.8	104.6 ± 11.4	0.109
Body fat mass (kg)			28.1 ± 11.4	29.4 ± 12.4	26.9 ± 10.5	0.228
AST above threshold			40.6 (65)	39.0 (30)	42.2 (35)	0.680
ALT above threshold			59.4 (95)	57.1 (44)	61.4 (51)	0.580
GGT above threshold			56.9 (87)	58.9 (43)	55.0 (44)	0.626
Type 2 diabetes mellitus (%)			41.0 (66)	45.5 (35)	36.9 (31)	0.271
High blood pressure (%)			58.4 (94)	66.2 (51)	51.2 (43)	0.053
Dyslipidemia (%)			59.6 (96)	57.1 (44)	61.9 (52)	0.538
Metabolic Syndrome (%)			50.3 (81)	57.1 (44)	44 (37)	0.097

Data presented as percentage (n) or mean ± SD; * *p*-value for comparison between MAFLD without alcohol and MAFLD with alcohol (independent samples T-test for continuous variables; Chi-Squared test for categorical variables); BMI—body mass index; AST—aspartate aminotransferase; ALT—alanine aminotransferase; GGT—gamma glutamyl transferase.

**Table 2:** Dietary intake in MAFLD patients.

	MAFLD Patients (*n* = 161)	MAFLD without Alcohol (*n* = 77)	MAFLD with Alcohol (*n* = 84)	*p*-Value *
Total energy intake (g/day)	1868 ± 415	1777 ± 381	1945 ± 429	0.013
Total fat intake (g/day)	74.2 ± 19.0	74.6 ± 18.4	73.9 ± 19.6	0.861
Saturated fat intake (g/day)	21.4 ± 6.3	21.3 ± 6.7	21.5 ± 6.1	0.687
MUFA intake (g/day)	34.3 ± 9.5	34.7 ± 9.3	33.9 ± 9.7	0.828
PUFA intake (g/day)	12.6 ± 3.9	12.5 ± 13.9	12.6 ± 3.9	0.575
Total protein intake (g/day)	83.2 ± 18.6	83.6 ± 18.3	83.1 ± 18.9	0.719
Total CHO intake (g/day)	209.8 ± 56.8	201.6 ± 56.0	216.7 ± 56.8	0.096
Sugar (g/day)	89.9 ± 37.5	86.2 ± 39.7	92.9 ± 35.5	0.094
Fiber intake (g/day)	19.2 ± 6.4	18.9 ± 6.5	19.5 ± 6.3	0.425

Data presented as mean ± SD; * *p*-value for comparison between MAFLD without alcohol and MAFLD with alcohol (independent samples T-test) MUFA—monounsaturated fatty acids; PUFA—polyunsaturated fatty acids; CHO—carbohydrates.

## Data Availability

The data presented in this study are available on request from the corresponding author. The data are not publicly available due to restrains on the consent provided by participants on the use of confidential data.
